# First-in-man phase I trial of two schedules of the novel synthetic tetrahydroisoquinoline alkaloid PM00104 (Zalypsis) in patients with advanced solid tumours

**DOI:** 10.1038/bjc.2012.99

**Published:** 2012-04-10

**Authors:** T A Yap, H Cortes-Funes, H Shaw, R Rodriguez, D Olmos, R Lal, P C Fong, D S Tan, D Harris, J Capdevila, C Coronado, V Alfaro, A Soto-Matos, C Fernández-Teruel, M Siguero, J M Tabernero, L Paz-Ares, J S de Bono, J A López-Martin

**Affiliations:** 1 Drug Development Unit, Royal Marsden NHS Foundation Trust, Downs Road, Sutton SM2 5PT, UK; 2 Division of Clinical Studies, The Institute of Cancer Research, Cotswold Road, Sutton SM2 5NG, UK; 3 Department of Medical Oncology, Hospital Universitario 12 de Octubre, Avenida Córdoba S/N, Madrid 28026, Spain; 4 Department of Medical Oncology, Vall d'Hebron University Hospital, Universitat Autònoma de Barcelona, Passeig de la Vall d’Hebron 119-129, Barcelona 08035, Spain; 5 PharmaMar, Clinical R&D, Avenida De los Reyes, 1 Pol. Ind. La Mina-Norte 28770-Colmenar Viejo, Madrid, Spain

**Keywords:** cytotoxic, novel marine-derived compound, phase I, PM00104

## Abstract

**Background:**

PM00104 binds guanines at DNA minor grooves, impacting DNA replication and transcription. A phase I study was undertaken to investigate safety, dose-limiting toxicities (DLTs), recommended phase II dose (RP2D), pharmacokinetics (PKs) and preliminary antitumour activity of PM00104 as a 1- or 3-h infusion three-weekly.

**Methods:**

Patients with advanced solid tumours received PM00104 in a dose escalation trial, as guided by toxicity and PK data.

**Results:**

A total of 47 patients were treated; 27 patients on the 1-h schedule (0.23–3.6 mg m^−2^) and 20 patients on the 3-h schedule (1.8–3.5 mg m^−2^). Dose-limiting toxicities comprised reversible nausea, vomiting, fatigue, elevated transaminases and thrombocytopenia, establishing the 1-h schedule RP2D at 3.0 mg m^−2^. With the 3-h schedule, DLTs of reversible hypotension and neutropenia established the RP2D at 2.8 mg m^−2^. Common PM00104-related adverse events at the RP2D comprised grade 1–2 nausea, fatigue and myelosuppression. In both schedules, PKs increased linearly, but doses over the 1-h schedule RP2D resulted in higher than proportional increases in exposure. A patient with advanced urothelial carcinoma had RECIST shrinkage by 49%, and three patients had RECIST stable disease ⩾6 months.

**Conclusion:**

PM00104 is well tolerated, with preliminary evidence of antitumour activity observed. The 1-h 3-weekly schedule is being assessed in phase II clinical trials.

PM00104 (Zalypsis, PharmaMar, Madrid, Spain) is a novel marine-derived synthetic tetrahydroisoquinoline alkaloid, which is structurally related to jorumycins (isolated from the Pacific nudibranch *Jorunna funebris*; Rinehart *et al*, 1990; Fontana *et al*, 2000) and renieramycins (from marine sponges; Figure 1; Oku *et al*, 2003). PM00104 showed potent cytotoxic activity against a wide range of human tumour cell lines, with IC_50_ values in the low nanomolar range (Leal *et al*, 2009). It has also demonstrated antitumour effects in multiple tumour xenograft mouse models, including prostate, gastric, pancreatic, bladder, breast, gastric and hepatocellular cancers (Elices *et al*, 2005a; Greiner *et al*, 2007; LePage *et al*, 2007a; Guillen *et al*, 2009), as well as multiple myeloma (Ocio *et al*, 2009).

PM00104 exerts its antitumour effects through the covalent modification of guanines in the DNA minor groove, thereby affecting DNA replication and transcription (Guirouilh-Barbat *et al*, 2009; Leal *et al*, 2009). DNA adducts are transformed into DNA double-strand breaks at nanomolar concentrations of PM00104 (Herrero *et al*, 2007), as detected by an increased proportion of cells with abundant *γ*H2AX foci formation in solid tumour and multiple myeloma cell lines (Guirouilh-Barbat *et al*, 2009; Leal *et al*, 2009; Ocio *et al*, 2009). DNA damage induced by PM00104 results in S-phase cell cycle arrest in various solid tumour cell lines (Magro *et al*, 2007; Leal *et al*, 2009) and activates DNA replication checkpoints. PM00104 caused tumour cells to accumulate in S-phase and induced apoptotic cell death, which was dependent on caspase activity, resulting in cleavage of poly (ADP-ribose) polymerase-1 and cytokeratin 18 (Leal *et al*, 2009; Ocio *et al*, 2009).

In preclinical studies, PM00104 given by intravenous injection produced toxicological effects primarily affecting the bone marrow, reticuloendothelial system and gastrointestinal tract in dogs and rats, as well as the liver and injection site. The gastrointestinal, bone marrow and lymphoid system alterations were completely reversible 2–3 weeks after the last drug administration (Yin *et al*, 2006).

The elimination kinetics of intravenous PM00104 in animals followed a biexponential profile, with calculated terminal half-lives of ∼7 and 10 h for mice and dogs, respectively, indicating a relatively slow plasma clearance (Yin *et al*, 2005a). The volume of distribution at steady state (*V*_ss_) was greater than plasma volume for all animal species tested, suggesting extensive extravascular distribution (Yin *et al*, 2006). *In vitro* metabolism studies of PM00104 demonstrated extensive hepatic metabolism; PM00104 mainly undergoes microsomal metabolism, with plasma protein binding >99%.

Given its preclinical antitumour activity and acceptable pharmacokinetic (PK) and toxicity profile in preclinical studies, a first-in-man, open-label, phase I study of PM00104 was undertaken. The primary objectives were to assess the safety and tolerability of PM00104, determine dose-limiting toxicities (DLTs) and establish the recommended phase II dose (RP2D) for PM00104 as a 1- or 3-h 3-weekly intravenous infusion. The secondary objectives were to characterise the PK and preliminary anticancer activity for these two 3-weekly schedules of PM00104.

## Materials and methods

This phase I study was conducted at the Royal Marsden NHS Foundation Trust (Sutton, UK), Hospital Universitario 12 de Octubre (Madrid, Spain) and Vall d'Hebron University Hospital (Barcelona, Spain) in accordance with the Declaration of Helsinki and the ICH Harmonised Tripartite Guideline for Good Clinical Practice and approved by the relevant regulatory authorities and independent ethics committees.

### Inclusion criteria

The study inclusion criteria were as follows: ⩾18 years with histopathological or cytological confirmation of cancer not amenable to conventional treatments; written informed consent; the Eastern Cooperative Oncology Group performance status ⩽2; life expectancy ⩾3 months; no residual toxicities from previous therapies (excluding alopecia and ⩽grade 1 (G1) peripheral neuropathy); adequate bone marrow, renal and hepatic function. Left ventricular ejection fraction (LVEF) ⩾50% was required. Exclusion criteria are detailed in the Supplementary Methods.

### Exclusion criteria

Patients were excluded if they had evidence of progressive central nervous system metastases or any symptomatic brain or leptomeningeal metastases; increased cardiac risk or other relevant clinical conditions such as active infection or uncontrolled endocrine diseases. Radiotherapy, chemotherapy, hormonal or biological therapies within 4 weeks before administration of PM00104 (8 weeks for extensive radiotherapy and 6 weeks for mitomycin C or nitrosurea therapy) were not permitted. Patients were also excluded if they were pregnant or breast-feeding, or if appropriate contraceptive measures were not being used.

### Drug administration

PM00104 was provided as a powder concentrate in 2.5 mg vials and reconstituted in 50–100 ml of 5% dextrose or saline solution for injection. PM00104 was initially administered as a 1-h intravenous infusion 3 weekly. A second schedule in which the duration of infusion was increased from 1 to 3 h was also planned *a priori* as an alternative strategy if toxicity due to PM00104-related maximum plasma concentration (*C*_max_), or if a more than proportional increase in *C*_max_ was observed during dose escalation with the 1-h schedule. The starting dose for the 1-h 3-weekly schedule was 0.23 mg m^−2^, that is, one-tenth of the maximum tolerated dose found for the most sensitive rodent species (mouse) in animal toxicology studies. The starting dose for the 3-h 3-weekly schedule was 1.8 mg m^−2^ (one dose level below the RP2D for the 1-h 3-weekly regimen). In both schedules, treatment was continued until discontinuation criteria were met, including disease progression, unacceptable toxicity or withdrawal of consent. Further details on DLT definitions and dose escalation design can be found in the Supplementary Methods.

### Study assessments

Assessments before, during and after study treatment included a medical history, physical examination, electrocardiogram and echocardiogram, haematology and chemistry profile, clotting studies and relevant scanning to evaluate tumour status. Toxicity was assessed at baseline and during treatment. All adverse events (AEs) and laboratory variables were graded according to the National Cancer Institute Common Terminology Criteria for Adverse Events v.3.0 (National Cancer Institute, 2006). As preclinical repeat-dose studies in rats had shown mortality related to cardiac degeneration, haemorrhage and mineralisation in some animals treated at the maximum tolerated doses in repeated cycles, a central independent cardiological assessment (including measurement of troponin I, standard ECG and evaluation of left ventricular ejection fraction by echocardiography) was carried out in phase I trials. The objective antitumour response was graded according to RECIST v.1.0 (Therasse *et al*, 2000) based on tumour size measurements made every two cycles.

### Dose-limiting toxicities and dose escalation

Dose-limiting toxicities were determined in the first treatment cycle and defined as follows: grade 4 neutropenia >5 days; grade 4 neutropenia associated with fever (⩾38 °C) or infection; grade 4 thrombocytopenia, and any other grade 3/4 PM00104-related non-haematological toxicity, except for nausea and/or vomiting unless they occurred despite optimal medical management; delay exceeding 2 consecutive weeks due to persistent drug-related toxicity; troponin I increase ⩾0.1 ng ml^−1^ with evidence of cardiac damage by echocardiogram or electrocardiogram, and decrease in LVEF >20% from baseline and/or LVEF <50%.

In the 1-h schedule, the dose of PM00104 was escalated by increments of 100% (50% if grade 2 toxicity occurred, and 25% if grade 3 or 4 toxicity occurred), while in the 3-h schedule, in view of the higher starting dosing, doses were escalated by increments of 25%. Dose escalation followed a 3+3 design; cohort expansion to six patients was required if one DLT was reported, and dose escalation stopped if two DLTs were observed. The RP2D was defined as the highest dose in which no >1 of a cohort of six patients had a DLT in the first cycle.

### Pharmacokinetics

Blood samples for PK analyses were collected on day 1 of cycles 1 and 2 at the following time-points: pre-dose, 10 min before the end of the infusion, and 0.25, 0.5, 1, 1.5, 2, 3, 4, 5 and 7 h post infusion. Additional samples were collected on days 2, 3, 4, 6, 8, 11 and 15. Blood samples were centrifuged at 2500 **g** for 15 min at 4 °C, and plasma was separated, placed in polypropylene tubes and stored at −20 °C until analysis. The concentrations of PM00104 in plasma samples were measured using a validated high-performance liquid chromatograph system coupled with electrospray ionisation tandem mass spectrometry (LC/MS-MS method; Yin *et al*, 2005b). Percentage coefficient of variation at the lower limit of quantification (LLOQ) varied from 2.26–14.69. Lower limit of quantification was ∼15 pg ml^−1^ in plasma. Non-compartmental PK parameters were calculated using WinNonLin version 5.2 (Pharsight, Mountain View, CA, USA).

## Results

### Patient characteristics

A total of 47 patients were enroled between December 2004 and January 2010, with 27 and 20 patients treated with the 1- and 3-h schedules of PM00104, respectively (Table 1).

### Recommended phase II dose

#### PM00104 1-h schedule

The dose was increased between cohorts until the 3.6 mg m^−2^ cohort, where two of six patients experienced DLTs in the first cycle (Table 2). One patient with suprarenal carcinoma developed G3 fatigue, nausea and vomiting on the first day of treatment despite maximal medical management. Nausea and vomiting improved to G1 within 3 days, while fatigue improved to G1 within 15 days of onset. A second patient with metastatic colorectal carcinoma had a transient G3 transaminase increase and G4 thrombocytopenia on days 2 and 7 of treatment, respectively. The transaminases returned to baseline levels after 7 days, while thrombocytopenia resolved within 14 days. As the previous dose level (1.8 mg m^−2^) was not associated with any significant toxicity, an intermediate dose level (3.0 mg m^−2^) was explored. No DLTs occurred in an expanded cohort of nine patients, and hence 3.0 mg m^−2^ was established as the RP2D of PM00104 on the 1-h 3-weekly schedule.

#### PM00104 3-h schedule

One DLT occurred at the first dose level (1.8 mg m^−2^) and two at the fifth dose level of 3.5 mg m^−2^ (Table 2). The patient with the DLT at 1.8 mg m^−2^ was a patient with metastatic NSCLC, who developed G3 hypotension on day 2 of PM00104. All cardiac assessments were normal and the DLT resolved within 2 days with intravenous fluids. The patient developed G2 vomiting after the end of his PM00104 infusion on day 1 and was also on regular antihypertensive medications, which could have contributed to the hypotension. No further DLTs were observed in the 1.8 mg m^−2^ cohort, and dose escalation continued with no DLTs until 3.5 mg m^−2^.

At this dose of 3.5 mg m^−2^ of PM00104, in the initial cohort of three patients, one patient with metastatic urothelial carcinoma developed G3 hypotension on day 2 of treatment associated with nausea, vomiting and diarrhoea, which resolved within 1 day. Another patient with melanoma developed G3 transaminase elevation and G2 hypotension due to Group A *Streptococcus* toxic shock syndrome, which resolved within 5 days of onset. As the relationship of these AEs to PM00104 in both patients was unclear, the 3.5 mg m^−2^ cohort was expanded to include three additional patients. Two of these subsequent three patients experienced DLTs; a patient with metastatic colorectal carcinoma had an asymptomatic and reversible G3 hypotension on day 2 of PM00104. He was treated with intravenous fluids with resolution of the DLT within 3 days without sequelae. A second patient with urothelial carcinoma developed G4 neutropenia on day 11 of treatment, which resolved after 7 days.

In view of the similar toxicities and the absence of an advantage in the PK exposure of PM00104 with the 3-h infusion, it was concluded that the 3-h infusion did not have any added benefit over the 1-h infusion. Furthermore, prolongation of infusion duration did not allow a higher PM00104 dose to be reached. Hence, the RP2D for this schedule was declared at 2.8 mg m^−2^.

### Toxicity profile at the RP2D

Most drug-related AEs or laboratory abnormalities (Table 3) experienced by patients receiving PM00104 at the RP2D of 3.0 mg m^−2^ for the 1-h schedule and 2.8 mg m^−2^ for the 3-h schedule were mild or moderate.

#### PM00104 1-h schedule

The most common AEs related to PM00104 treatment were nausea (*n*=6), fatigue (*n*=5) and injection site phlebitis (*n*=4). No treatment-related AE reached G4. Frequent haematological abnormalities irrespective of the grade or relationship with treatment included anaemia (*n*=8), neutropenia (*n*=6) and leucopenia (*n*=4). Grade 3/4 neutropenia appeared in four patients at a median of 15 days after dosing, reaching the nadir on the same day and lasting a median of 7 days. Frequent biochemical abnormalities included raised ALP (*n*=4) and transaminase levels (*n*=3). Only one patient had grade 3 AST increase, with the onset and peak of the event on day 3 of cycle 2. No data on AST recovery were available, as this patient died due to disease progression.

#### PM00104 3-h schedule

The most common AEs related to PM00104 treatment were constipation, nausea and vomiting (*n*=3). No treatment-related AE reached G3 or G4, or had consequences on treatment. The most frequent haematological abnormalities irrespective of grade or relationship with treatment were anaemia and neutropenia. Only one patient had severe neutropenia, reaching grade 4, with the onset and nadir of the event on day 18, and lasting 3 days. Frequent biochemical abnormalities were ALP and ALT increases (*n*=2). No severe biochemical abnormalities occurred at the RP2D.

### PM00104 administration and tolerability

#### PM00104 1-h schedule

A total of 73 cycles (median of 2 cycles per patient; range, 1–7) were administered from 0.23 to 3.6 mg m^−2^. About 10.9% of infusions were delayed due to PM00104-toxicities (median delay of 7 days; range, 3–11 days), with transient G1–G3 neutropenia and G2–G3 fatigue most commonly observed. At the 1-h schedule RP2D of 3.0 mg m^−2^, the median dose intensity was 1 mg m^−2^ per week and the median relative dose intensity was 100% (range, 54.2–100.5%).

#### PM00104 3-h schedule

In all, 58 cycles (median of 2 cycles per patient; range, 1–8) were administered from 1.8 to 3.5 mg m^−2^. About 13.2% of infusions were delayed due to PM00104-toxicities (median delay of 7 days; range, 5–8 days), with transient G2–G3 neutropenia and G2 fatigue most commonly observed. At the 3-h schedule RP2D of 2.8 mg m^−2^, the median dose intensity was 0.93 mg m^−2^ per week and the median relative dose intensity was 100% (range, 85.8–100%).

### Cardiological assessment

Non-clinically relevant increases in cardiac troponin I >ULN were observed in 5 of 27 patients treated with the 1-h schedule and in 6 of 20 patients treated with the 3-h 3-weekly schedule. There was no clear pattern for the onset and duration of raised troponin I levels following PM00104 treatment. These increases in cardiac troponin I levels were not associated with any concomitant symptoms or alterations in electrocardiograms and echocardiograms. A review of electrocardiograms and echocardiograms by an independent board-certified cardiologist found no evidence of cardiac toxicity. Cardiac MRI scans undertaken in two patients treated within the 3-h 3-weekly schedule confirmed no evidence of drug-induced cardiac damage.

In one patient in the 1-h infusion cohort, cardiac abnormalities comprising G2 T-wave inversion on electrocardiogram was considered dose-limiting. Full cardiac workup was undertaken, including a coronary angiogram and echocardiogram, none of which revealed significant findings that could account for the electrocardiogram changes.

### Pharmacokinetics

**Figure 2:**
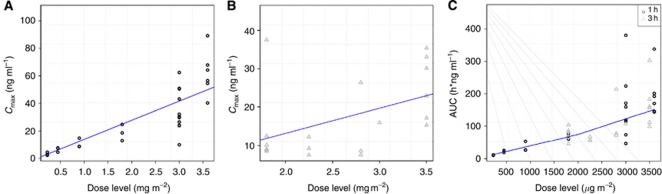
Pharmacokinetics: *C*_max_*vs* dose level. (**A**) 1-h 3-weekly schedule; (**B**) 3-h 3-weekly schedule and (**C**) dose level *vs* AUC by infusion length.

Relevant PK data for PM00104 administered in 1- and 3-h schedules are shown in Table 4. With both schedules, area under the curve (AUC) and *C*_max_ increased dose proportionally up to the RP2D (Figure 2).

Weight and BSA were related to the *V*_ss_, but not to PM00104 clearance (Supplementary Figure 1). Gender and performance status did not have an impact on PK parameters. An increase in AST was directly related with longer half-life, while renal function did not affect PK parameters. The plasma proteins were inversely related with *V*_ss_: an increase in plasma proteins was related to a decrease in *V*_ss_. The analysis of the effect of PM00104 PK exposure on biochemical and haematological parameters (measured as the ratio of the worst level in cycle 1 to the baseline level of each parameter) showed that a longer half-life and increases in AUC and *C*_max_ were related with higher transaminases and bilirubin, and lower counts of neutrophils, platelets and white blood cells, whereas haemoglobin and renal function were not affected.

### Antitumour activity

**Figure 3:**
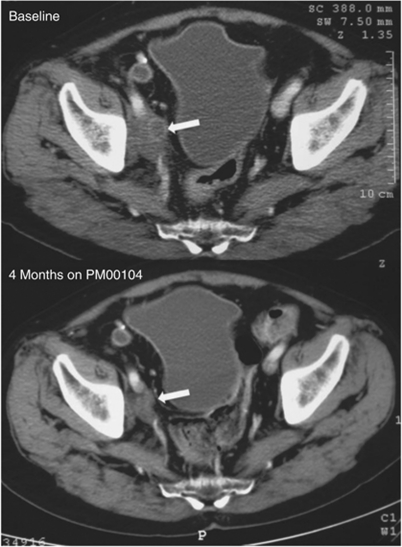
Pre- and post-treatment CT scans of the patient treated with four cycles of PM00104 3.5 mg m^−2^ 3-h 3-weekly and with partial response according to Response Evaluation Criteria in Solid Tumors (RECIST). These are CT scans from a 64-year-old male with metastatic urothelial cancer who had previously received cisplatin/gemcitabine, radiotherapy to the pelvis and carboplatin/paclitaxel. The maximum tumour shrinkage in the target lesion (right iliac lymph node) was 49.0% by RECIST measurements. However, disease progression was found in the brain after cycle 4, leading to drug discontinuation (time to progression of 4.2 months).

A total of 39 of 47 patients treated with both schedules of PM00104 were evaluable for antitumour efficacy assessments. Tumour shrinkage was observed in a patient with metastatic urothelial cancer (previous history of prostate adenocarcinoma) treated with 3.5 mg m^−2^ of PM00104 in a 3-h schedule. This heavily pretreated patient achieved RECIST tumour shrinkage of 49.0% after two cycles of PM00104 (Figure 3). However, the patient developed a symptomatic brain metastasis 7 days after his post-cycle 2 CT evaluation. The patient temporarily discontinued PM00104 to receive whole-brain radiotherapy, but after acquiring appropriate Ethics Committee permission, proceeded to receive further cycles of PM100104. He eventually developed disease progression in his brain metastasis after cycle 4, leading to drug discontinuation after 4.2 months. Three other patients had RECIST disease stabilisation >6 months, including patients with advanced urothelial carcinoma (3.0 mg m^−2^; 1-h schedule), head and neck squamous cell carcinoma (3.0 mg m^−2^; 1-h schedule) and NSCLC (1.8 mg m^−2^; 3-h schedule), lasting 9.8, 8.6 and 6.1 months, respectively (Supplementary Table 1). All three patients had radiological disease progression before enrolment on study.

## Discussion

The primary objectives of this phase I study, to assess the safety and establish the RP2D of two schedules of PM00104 in patients with advanced solid tumours, were met. All DLTs observed were fully reversible. Dose-limiting toxicities observed with the 1-h schedule of PM00104 included G3 transaminitis, fatigue, nausea and vomiting and G4 thrombocytopenia (*n*=1 each). Dose-limiting toxicities with the 3-h schedule of PM00104 included G3 hypotension (*n*=2) and G4 neutropenia >5 days (*n*=1). These findings are consistent with preliminary results from other phase I trials evaluating other schedules of PM00104, in which myelosuppression, nausea and vomiting have also been observed (Soria *et al*, 2008; Capdevila *et al*, 2009), in keeping with this agent’s mechanism of action. For the 1-h 3-weekly schedule, the RP2D was established at 3.0 mg m^−2^. Prolongation of 3-weekly infusion duration from 1 to 3 h did not result in higher exposures of PM00104, with DLTs observed at a dose of 3.5 mg m^−2^. As a result, the dose declared as the RP2D for the 3-h 3-weekly schedule was 2.8 mg m^−2^.

Both schedules were generally well tolerated and associated with manageable and reversible toxicity. PM00104-related AEs at the RP2D for both schedules were mostly G1–G2, with the most frequently observed toxicities being transient nausea, vomiting and fatigue. As some infusion site reactions occurred with the 1-h 3-weekly schedule, the administration of PM00104 via central intravenous access during this study became mandatory in all clinical trials of PM00104. Furthermore, as PM00104 has a moderate emetogenic risk, primary prophylaxis utilising anti-emetics according to the American Society of Clinical Oncology guidelines (Kris *et al*, 2006) with dexamethasone and 5-HT3 antagonists was pursued. The most common G3–G4 haematological abnormalities with both schedules of PM00104 at the respective RP2Ds were transient and comprised neutropenia and leucopenia (1-h), and neutropenia alone (3-h).

In this phase I trial, isolated increments in cardiac troponin I levels were observed in a minority of patients (*n*=5, 1-h 3-weekly; *n*=6, 3-h 3-weekly), with no associated symptoms or alterations in electrocardiograms or echocardiograms. Data from this and other phase I studies of PM00104 (Soria *et al*, 2008; Capdevila *et al*, 2009) suggest that there is no evidence of acute cardiac damage in patients treated with PM00104. Troponin I was tested from the outset of this phase I trial because preclinical data in rats identified haemorrhage, necrosis and mineralisation of cardiac muscle. In other phase I studies of PM00104 (Soria *et al*, 2008; Capdevila *et al*, 2009), increases in troponin I were also observed, but no other cardiac studies were found to be abnormal. Serial ECGs, echocardiograms, cardiac MRI and coronary angiogram studies in patients remained normal, while LVEF was maintained within normal ranges during the trials. The long-term effects of such a rise in troponin I levels is currently unknown, but may suggest that elevations in troponin I levels observed in this small subset of patients receiving PM00104 may simply reflect a biochemical finding, rather than clinical sequelae of cardiac damage. A phase II study of PM00104 with a sub-analysis of troponin I using a 1-h 3-weekly schedule is currently ongoing.

Hypotension observed on this trial was transient, and no underlying drug-related pathophysiological mechanism has been established. In patients with symptoms including emesis and diarrhoea, dehydration may have had a role as all episodes reversed with intravenous rehydration. In a pooled analysis of 144 patients treated in four phase I single-agent trials of PM00104, 5% (*n*=8) had hypotension, although this was G3 in only three patients (2%). No other relevant cardiovascular events were reported concomitant with hypotension.

Non-compartmental analysis of the PK profile of PM00104 demonstrated dose proportionality of the main parameters (AUC and *C*_max_) up to the RP2D. A population PK analysis including data from this and other phase I studies (Perez-Ruixo *et al*, 2012) confirmed that PM00104 has linear PKs.

Three patients with advanced solid tumours had RECIST stable disease ⩾6 months. Furthermore, a patient with advanced urothelial carcinoma had a RECIST partial response, with tumour shrinkage by 49% after 2 months of treatment, indicating that PM00104 has antitumour activity. PM00104 is a large molecule that does not cross the blood brain barrier, which may explain the progression of a brain metastasis in this patient.

In conclusion, the primary objective of this phase I trial was met, and the RP2D was established as 3.0 mg m^−2^ for the 1-h PM00104 schedule and as 2.8 mg m^−2^ for the 3-h PM00104 schedule. In view of the well-tolerated toxicities, active PK profile and preliminary evidence of antitumour activity observed for PM00104 in this first-in-human study, multiple phase II clinical trials have now been initiated. Currently, PM00104 is being assessed in phase II studies undertaken in patients with advanced endometrial cancer, cervical cancer, multiple myeloma, advanced Ewing family of tumours and advanced urothelial cancer (http://www.clinicaltrials.gov).

## Figures and Tables

**Figure 1:**
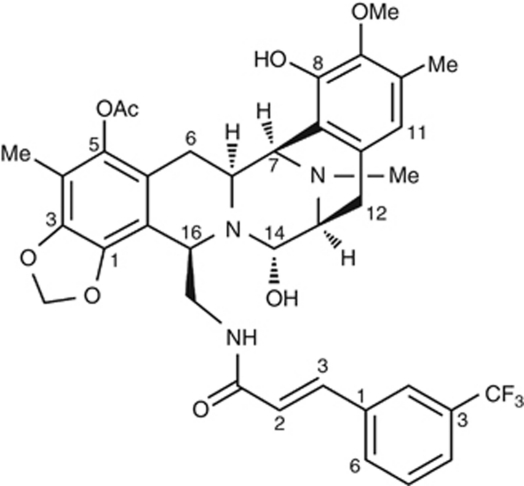
Structural formula of PM00104 (Zalypsis; C_37_H_38_F_3_N_3_O_8_).

**Table 1:** Patient characteristics (*n*=47)

	**1-h schedule (27 patients)**		**3-h schedule (20 patients)**	
	**No. of patients**	**%**	**No. of patients**	**%**
Male/female	19/8	70/30	15/5	75/25
				
Median age in years (range)	58 (39–79)		60 (20–74)	
				
*ECOG performance status*				
0	14	51.9	12	90.0
1	11	40.7	8	10.0
2	2	7.4	—	—
				
*Primary tumour type*				
NSCLC	5	18.5	3	15.0
Colorectal carcinoma	1	3.7	5	25.0
Soft tissue sarcoma	4	14.8	1	5.0
Osteosarcoma	2	7.4	2	10.0
Malignant melanoma	3	11.1	1	5.0
Urothelial carcinoma	1	3.7	3	15.0
Other	11^a^	40.8	5^b^	25.0
				
*Tumour stage*				
Metastatic	23	85.2	18	90.0
Locally advanced	4	14.8	2	10.0
				
*Sites of metastasis*				
Lung	19	70.4	10	50.0
Lymph nodes	12	44.4	9	45.0
Liver	7	25.9	7	35.0
Bone	5	18.5	2	10.0
Soft tissue	6	22.2	—	—
Retroperitoneal	—	—	2	10.0
Other	17^c^	62.9	6^d^	30.0
				
*Prior treatment*				
Surgery	17	63.0	13	65.0
Radiotherapy	11	40.7	7	35.0
Chemotherapy	27	100.0	20	100.0
				
*Number of prior chemotherapy regimens*				
Median	3		3	
Range	1–6		1–9	
<3	18	66.7	14	70.0
⩾3	9	33.3	6	30.0

Abbreviations: ECOG=Eastern Cooperative Oncology Group; NSCLC=non-small cell lung cancer. In both schedules, PM0014 was administered as an intravenous 3-weekly infusion.

a Pleural mesothelioma (*n*=3), neuroendocrine (*n*=2), head and neck (*n*=2), oesophageal, prostate, renal cell and suprarenal (*n*=1 each).

b Pancreas (*n*=2), breast, oesophageal and hepatocarcinoma (*n*=1 each).

c Abdomen, intra-abdominal, pancreas, pleura, spleen, suprarenal (*n*=2 each), oesophagus, pelvis, retroperitoneal, skin and one non-identified site (*n*=1 each).

d Esophagus, pancreas, pelvis, spleen, stomach and thorax (*n*=1 each).

**Table 2:** Dose escalation schema and DLTs

**Dose level**	**Dose (mg m^−2^)**	**No. of patients**	**No. of cycles**	**No. of patients with DLTs in cycle 1**	**Description of DLTs**
*1-h 3-weekly schedule*					
I	0.23	3	11	—	—
II	0.45	3	5	—	—
III	0.9	3	10	—	—
IV	1.8	3	7	—	—
V^a^	3.0	9	32	—	—
VI^b^	3.6	6	8	2	Grade 3 nausea Grade G3 vomiting (despite antiemetic treatment) Grade G3 fatigue Grade 3 transaminase increase Grade 4 thrombocytopenia
Total		27	73		

*3-h 3-weekly schedule*					
I	1.8	6	24	1	Grade 3 hypotension
II	2.3	3	10	—	
III^a^	2.8	4	8	—	
IV^c^	3.0	1	3	—	
V^b^	3.5	6	13	2	Grade 3 hypotension Grade 4 neutropenia (lasting>5 days)
Total		20	58		

Abbreviations: DLT=dose-limiting toxicity; RP2D=recommended phase II dose.

a RP2D on indicated schedule.

b Maximum tolerated dose.

c Cohort was discontinued once the 3-h schedule showed no advantage over the 1-h schedule (prolongation of infusion durations from 1 to 3 h did not allow a higher RP2D dose to be reached).

**Table 3:** Drug-related AE (⩾10% of patients) and laboratory abnormalities (haematological and biochemical) at the RP2D

	**3.0 mg m ^−2^(1-h schedule)**				**2.8 mg m ^−2^(3-h schedule)**			
	**Per patient (*n*=9)**		**Per cycle (*n*=32)**		**Per patient (*n*=4)**		**Per cycle (*n*=8)**	
*Drug-related AE (⩾10% of patients) at the RP2D*								
NCI-CTCAE grade	1–2	3–4^a^	1–2	3–4^a^	1–2	3–4	1–2	3–4
Abdominal pain	—	—	—	—	1	—	2	—
Anorexia	2	1	5	1	—	—	—	—
Constipation	—	—	—	—	3	—	5	—
Diarrhoea	—	1	2	1	1	—	2	—
Dizziness	1	—	2	—	—	—	—	—
Dyspepsia	—	—	—	—	1	—	2	—
Fatigue	3	2	7	3	2	—	2	—
Hypotension	1	—	1	—	—	—	—	—
Injection site phlebitis	4	—	6	—	—	—	—	—
Mucosal inflammation	1	—	1	—	—	—	—	—
Nausea	6	—	10	—	3	—	5	—
Oral candidiasis	2	—	2	—	—	—	—	—
Peripheral neuropathy	1	—	1	—	—	—	—	—
Pyrexia	—	—	—	—	1	—	1	—
Vomiting	2	—	3	—	3	—	5	—
Weight decreased	1	—	2	—	—	—	—	—
								
*Laboratory abnormalities (haematological and biochemical) at the RP2D*								
ALT increased	3	—	5	—	2	—	2	—
Amylase increased	2	—	2	—	1	—	2	—
Anaemia	8	—	28	—	4	—	7	—
ALP increased	4	—	8	—	2	—	3	—
AST increased	2	1	3	1	1	—	1	—
Creatinine increased	2	—	3	—	1	—	2	—
Leucopenia	2	2	7	2	1	—	2	—
Neutropenia	2	4	10	4	2	1	4	1
Thrombocytopenia	3	—	4	—	—	—	—	—
Total bilirubin increased	3	—	3	—	—	—	—	—

Abbreviations: AE=adverse event; NCI-CTCAE=National Cancer Institute Common Terminology Criteria for Adverse Events; RP2D=recommended phase II dose. In both schedules, PM00104 was administered as an intravenous 3-weekly infusion.

a Depicted are the numbers of patients/cycles with the specified AE. Only grade 3 was reached with the 1-h schedule.

**Table 4:** Non-compartmental PK parameters of PM00104: 1- and 3-h 3-weekly schedules

**Dose (mg m^−2^)**	**No. of patients**	***C*_max_ (ng ml^−1^)**	**AUC (ng h ml^−1^)**	**CL (l/h)**	***V*_ss_(l)**	**Terminal *t*_½_(h)**
*1-h schedule*						
0.23	3	3.31±1.18	10.17±0.84	40.2±3.9	660±62	17.2±3.5
0.45	3	6.66±1.76	21.83±3.75	38.9±11.2	682±185	19.3±3.4
0.9	3	10.76±3.42	34.91±15.37	51.2±19.1	783±315	15.8±2.9
1.8	3	18.76±5.90	67.99±8.63	49.9±11.8	868±268	17.5±2.0
3.0	9	35.17±16.28	155.50±99.76	48.0±29.3	1038±671	25.8±10.3
3.6	6	62.12±16.34	198.33±72.07	35.4±9.0	701±110	29.5±8.1

*3-h schedule*						
1.8	6	14.37±11.43	74.73±20.30	43.8±10.9	674±304	20.6±8.8
2.3	3	9.61±2.40	59.57±6.33	65.1±11.6	814±282	13.3±3.2
2.8	3^a^	14.12±10.61	120.85±80.36	57.3±29.9	1032±427	21.9±11.5
3.0	1	15.90	106.57	50.7	1024	26.1
3.5	6	25.63±8.45	168.67±73.12	44.5±16.8	749±171	26.0±8.6

Abbreviations: AUC=area under the curve; CL=clearance; Cmax=maximum plasma concentration; t½=half-life; Vss=volume of distribution in steady state. In both schedules, PM0014 was administered as an intravenous 3-weekly infusion. Results shown are mean±s.d. Data obtained during the first cycle.

a One patient was excluded from the analysis because of having an unclear PK pattern likely due to an erroneous sample collection procedure.
