# Supplementary material for: First-in-man phase I trial of two schedules of the novel synthetic tetrahydroisoquinoline alkaloid PM00104 (Zalypsis) in patients with advanced solid tumours
Source: Br J Cancer. 2012 Apr 10;106(8):1379–85. doi: 10.1038/bjc.2012.99 (PMC3326684; doi:10.1038/bjc.2012.99)
Supplement: Supplementary Information [file bjc201299x1.doc]

**SUPPLEMENTAL TABLES AND FIGURES**

**Supplemental Table 1.** Overall response data of patients

**Supplemental Figure 1A.** Pharmacokinetics: Clearance (L/h) vs Weight (kg)

**Supplemental Figure 1B.** Pharmacokinetics: Clearance (L/h) vs Body Surface Area (m^2^)

**Supplemental Figure 1C.** Pharmacokinetics: V_ss_ (l) vs Weight (kg)

**Supplemental Figure 1D.** Pharmacokinetics: V_ss_ (l) vs Body Surface Area (m^2^)

**Supplemental Table 1. Overall response data of patients**

| Dose level  **(mg/m^2^)** | **Age**  **(years)** | **Primary tumor** | **Prior treatment** | PM00104 | | |
| --- | --- | --- | --- | --- | --- | --- |
|  |  |  | **No. of regimens/best response** | **Cycles** | **Best response** | **TTP (months)** |
| **PM00104 1-hour schedule** | | | | | | |
| **0.23** | 77 | Prostate adenocarcinoma | 9 / PD | 5 | SD | 3.4 |
| **3.0*** | 76 | Head and neck carcinoma | 2 / SD | 6 | SD | 8.6 |
|  | 61 | Urothelial carcinoma | 4 / SD | 6 | SD | 9.8 |
|  | 73 | Pleural mesothelioma | 2 / SD | 7 | SD | 4.0 |
| **PM00104 3-hour schedule**** | | | | | | |
| 1.8 | 57 | Osteosarcoma | 4 / SD | 6 | SD | 4.2 |
|  | 70 | NSCLC | 3 / PD | 8 | SD | 6.1 |
| 2.3 | 74 | NSCLC | 6 / SD | 6 | SD | 4.4 |
| 3.0 | 67 | Colorectal adenocarcinoma | 3 / PD | 3 | SD | 3.4 |
| 3.5 | 64 | Urothelial carcinoma | 2 / SD | 4 | PR^#^ | 4.2 |
| In both schedules, PM0014 was administered as an intravenous 3-weekly infusion.  * RP2D, recommended phase II dose.  **Of note, a 42-year-old male with suprarenal carcinoma treated with PM00104 3.6mg/m^2^ 1-hour 3-weekly was non-evaluable (only one RECIST evaluation while on treatment), but had a TTP of 11.5 months.  ^#^ This patient had a brain metastasis observed after cycle 2, however the only target lesion was found reduced after this cycle  Abbreviations: NSCLC, non-small cell lung cancer; PR, partial response; RECIST, Response Evaluation Criteria in Solid Tumors; SD, stable disease; TTP, time to progression. | | | | | | |

**Supplemental Figure 1A**


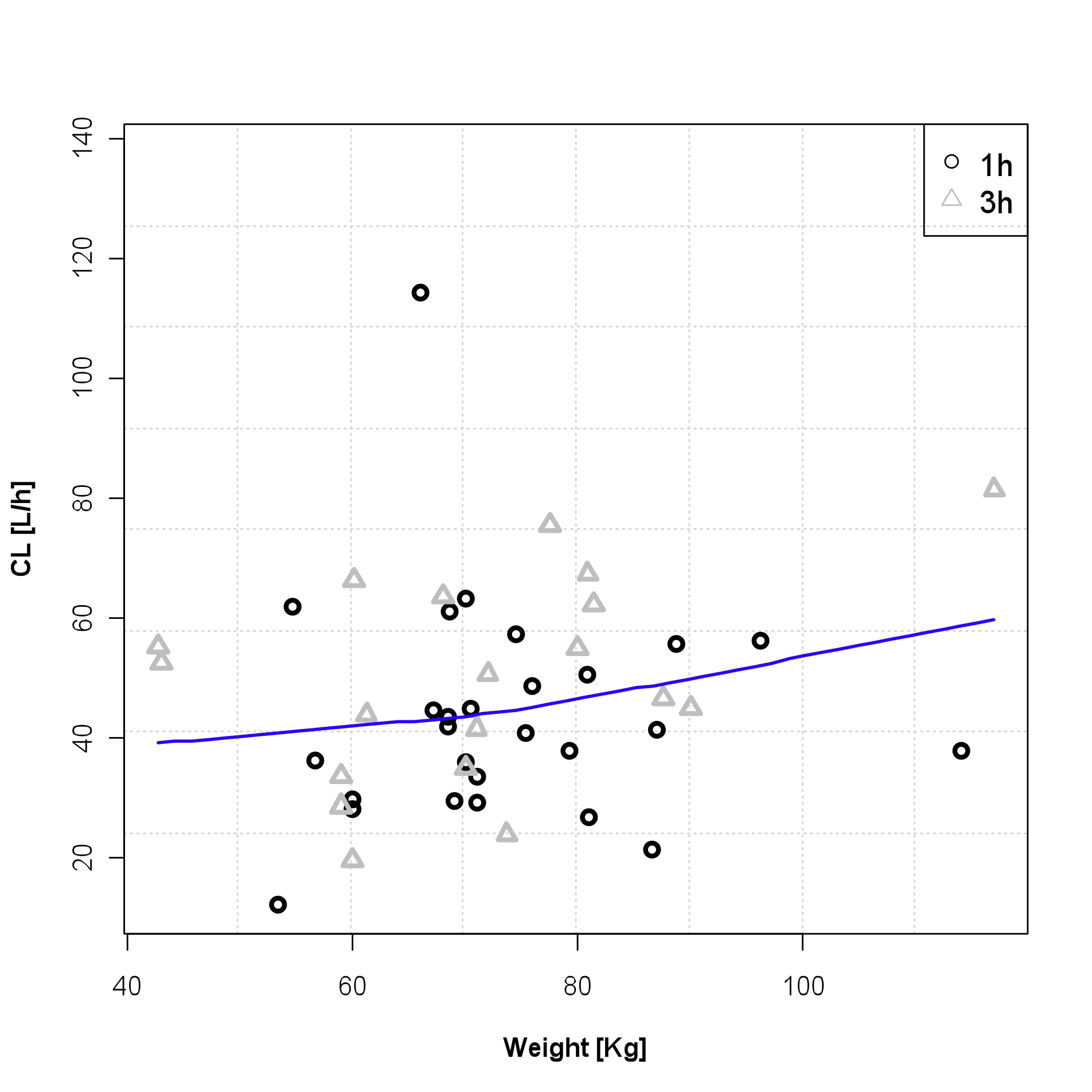


**Supplemental Figure 1B**


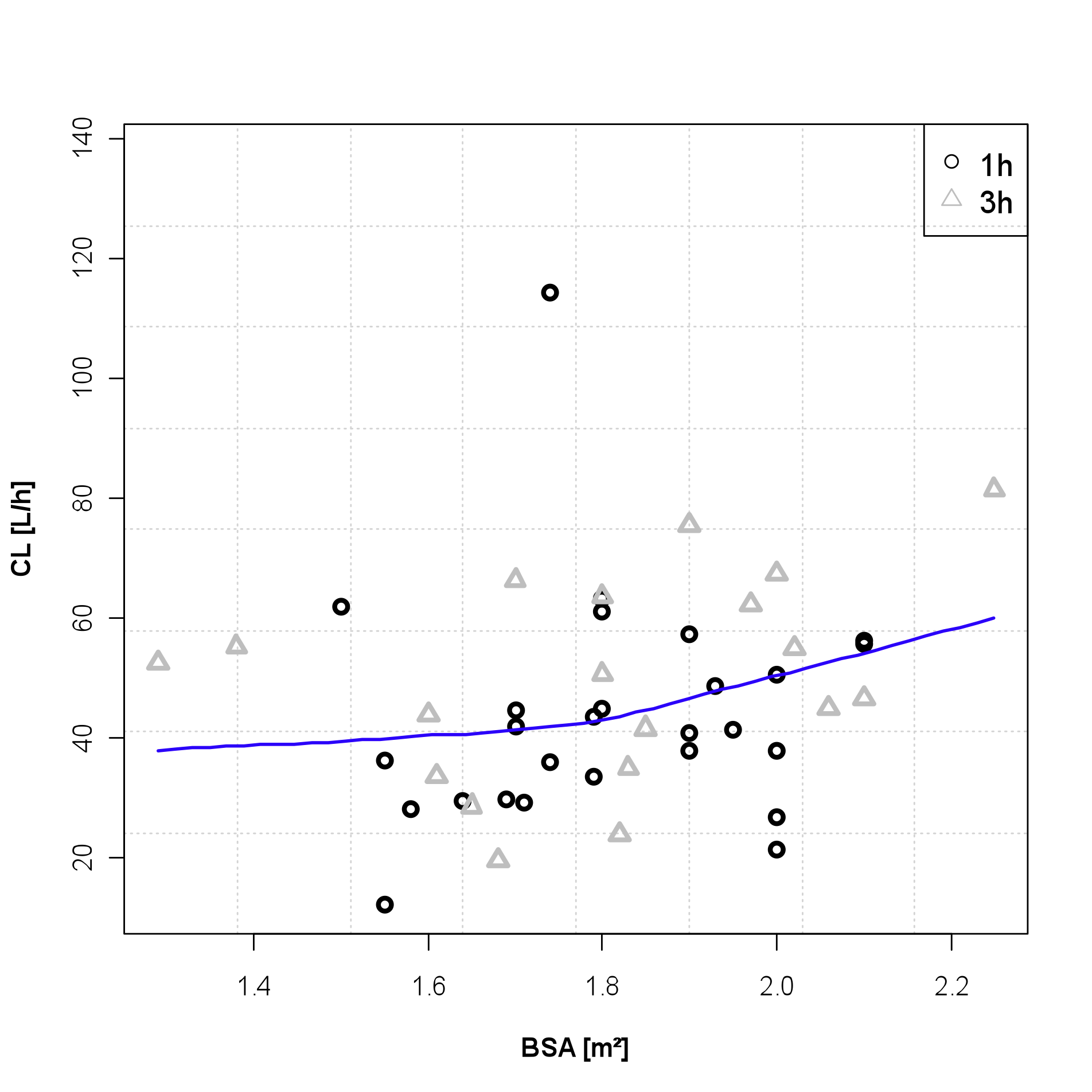


**Supplemental Figure 1C**

**
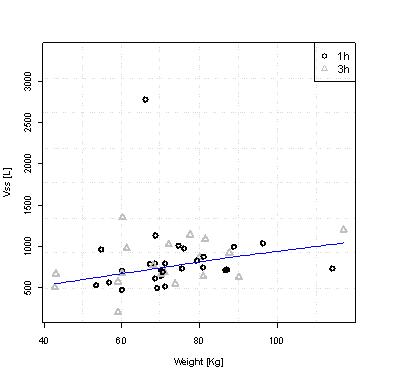
**

**Supplemental Figure 1D**

**
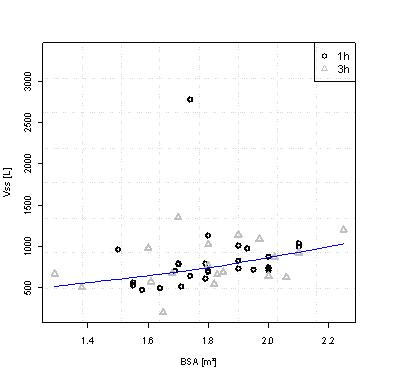
**
